# Active DNA demethylation—The epigenetic gatekeeper of development, immunity, and cancer

**DOI:** 10.1002/ggn2.10033

**Published:** 2020-11-27

**Authors:** Rahul Prasad, Timothy J. Yen, Alfonso Bellacosa

**Affiliations:** ^1^ Cancer Epigenetics and Cancer Biology Programs, Fox Chase Cancer Center Philadelphia Pennsylvania USA

**Keywords:** AID, APOBEC, DNA deamination, DNA demethylation, DNA methylation, TDG, TET

## Abstract

DNA methylation is a critical process in the regulation of gene expression with dramatic effects in development and continually expanding roles in oncogenesis. 5‐Methylcytosine was once considered to be an inherited and stably repressive epigenetic mark, which can be only removed by passive dilution during multiple rounds of DNA replication. However, in the past two decades, physiologically controlled DNA demethylation and deamination processes have been identified, thereby revealing the function of cytosine methylation as a highly regulated and complex state—not simply a static, inherited signature or binary on‐off switch. Alongside these fundamental discoveries, clinical studies over the past decade have revealed the dramatic consequences of aberrant DNA demethylation. In this review we discuss DNA demethylation and deamination in the context of 5‐methylcytosine as critical processes for physiological and physiopathological transitions within three states—development, immune maturation, and oncogenic transformation; and we describe the expanding role of DNA demethylating drugs as therapeutic agents in cancer.

## Graphical abstract

Thymine DNA glycosylase (TDG) “clears the cytosine bases” through base excision repair. DNA methyltransferases “start the ball game” by methylating cytosine at the 5‐carbon position to produce 5‐methylcytosine (5mC), which then serves as a substrate for oxidation or deamination. Left panel: In the case of oxidation, ten‐eleven translocation (TET) enzymes sequentially oxidize 5mC, first to 5‐hydroxymethylcytosine (5hmC), then to 5‐formylcytosine and finally 5‐carboxylcytosine. Both of the latter oxidation products are substrates for base excision by TDG. Right panel: In the case of deamination, 5mC is deaminated by AID/APOBEC family enzymes to thymine (T), creating a T:G mismatch—the nominal substrate for TDG. Similarly, 5hmC could possibly be deaminated to 5‐hydroxymethyluracil (5hmU), another TDG substrate. In addition to 5mC, TET enzymes can also oxidize thymine to 5hmU. Intermediates formed by either process, in particular the abasic site (AP) can be enzymatically targeted by downstream base excision repair activities (AP endonuclease, DNA polymerase β, DNA ligase). Of note, methyl‐binding domain‐4 and SMUG1 are the only other enzymes with known T:G and hmU:G base excision function, respectively.

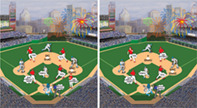

## 1 INTRODUCTION

DNA methylation—the addition of a methyl group at the five position of cytosine (C) in DNA to form 5‐methylcytosine (5mC)—is a critical process in the regulation of gene expression that affects multiple cellular functions and is ultimately responsible for significant organ‐level effects in mammals. DNA methylation was first discovered in the context of development, with the vast majority found at palindromic CpG dinucleotides. The presence or absence of these marks within gene promoters and enhancers have since been shown to influence progenitor and stem cells across the developmental spectrum. The hematopoietic system is one of the best examples of DNA methylation influence, as changes accompany every step of stem cell functional development, and ultimately affect the commitment, progression, and differentiation of the erythroid, myeloid, and lymphoid lineages. Given its significance in regulating cellular states and functions, defects in CpG methylation are responsible for a plethora of developmental problems.

It is tempting to consider genomic DNA methylation patterns, that is, the DNA methylome, as merely repressive marks, which are only detrimental when ectopically present or absent at particular genes of interest. However, disruptions of the DNA methylome in cancer suggest higher level functions preceding regulation of gene expression.^1^, ^2^, ^3^ One such disruption is the genome‐wide hypomethylation of LINE‐1 in cancer, a phenomenon seen in multiple tissue types.^4^, ^5^, ^6^, ^7^ The function of DNA methylation as a regulator of cellular proliferation and differentiation naturally implies a role in oncogenesis, which has indeed been amply corroborated. Evidence continues to grow in identifying both developmentally relevant and cancer‐specific gene expression changes that are driven by aberrations in the DNA methylome.

Only recently has it become clear that the DNA methylome is tightly and dynamically regulated, with both “writers” and “erasers” modulating epigenetic marks in opposite directions via enzymatic activity. DNA methyltransferases (DNMTs) were identified as a class of enzymes, not only conserving or maintaining methylated sites following DNA replication (DNMT1), but also capable of de novo placement of methylation sites (DNMT3A and DNMT3B). The significant medical result of uncovering the process of maintenance and de novo methylation was the discovery of two cytidine analogs, 5‐azacytidine (azacitidine), and its deoxy derivative 5‐aza‐2′‐deoxycytidine (decitabine). These two analogs were originally developed as anticancer antimetabolites, and can irreversibly inhibit DNMTs through covalent binding, ultimately leading to hypomethylation.

While the inhibition of DNMT enzymes induces a gradual, accumulated reduction in methylation, a physiological system of removing cytosine methylation has long been suspected, but remained controversial until approximately 10 years ago. The identification of dioxygenases of the ten‐eleven translocation (TET) family and of the DNA repair enzyme thymine DNA glycosylase (TDG) as the main enzymes responsible for active DNA demethylation in mammals confirmed this suspicion. Over the past decade, much progress has been made in delineating the mechanism of DNA demethylation in both biochemical and clinical contexts. A comprehensive view of the current data suggests that DNA demethylation plays a critical role in physiological and physiopathological transitions, with expanding potential as a therapeutic target.

## 2 MECHANISMS OF DNA DEMETHYLATION

**FIGURE 1:**
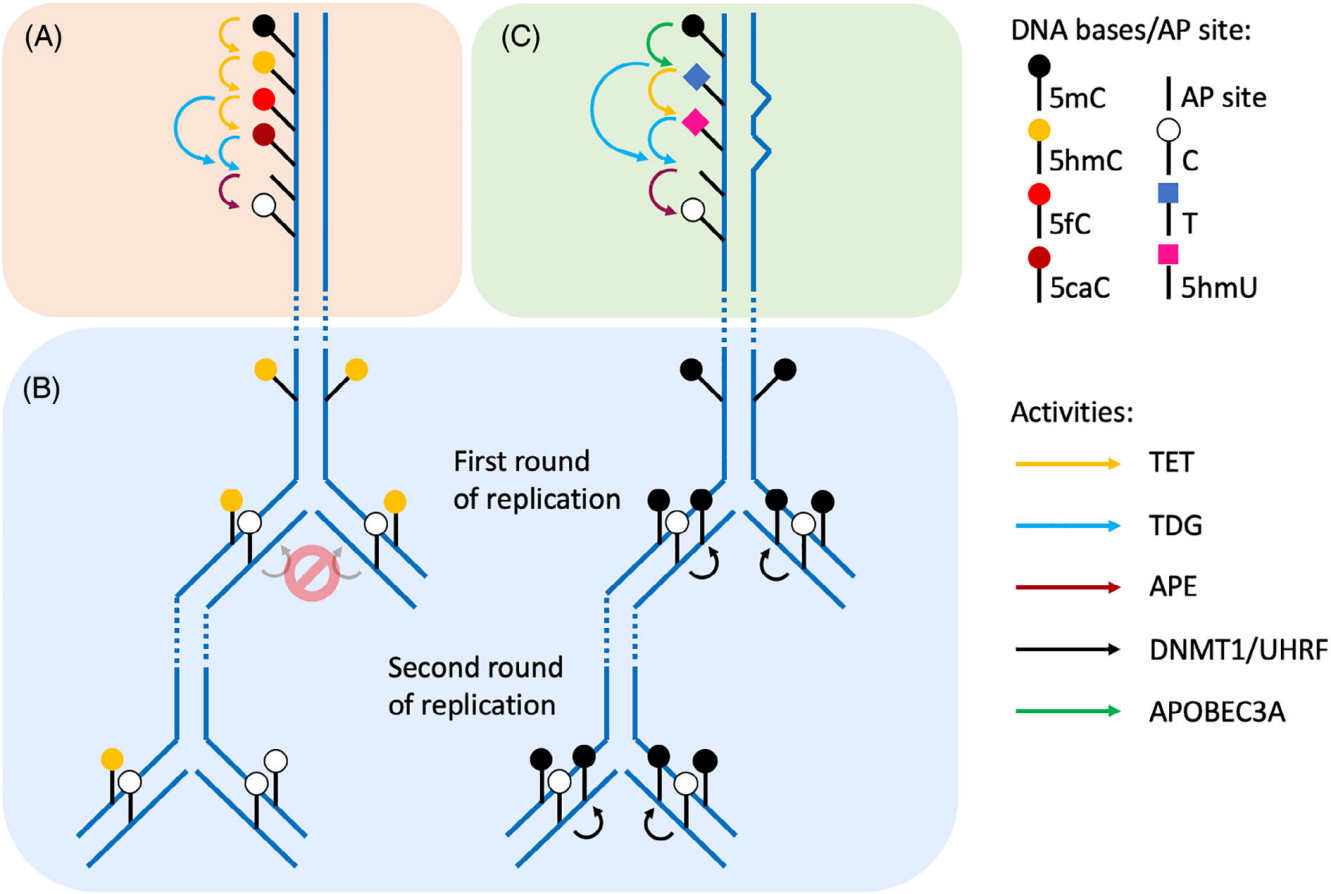
Pathways of DNA demethylation through oxidation or deamination. Bases are signified by colored shapes (circles = cytosine variants, square = thymine/hydroxymethyluracil), the stem signifying the deoxyribose moiety. A, *Replication‐independent DNA demethylation*: ten‐eleven translocation (TET) enzymes oxidize 5mC (black) to 5hmC (orange) and then sequentially further oxidize to 5fC (red) and 5caC (burgundy). All products are stable, and the latter two are substrates for thymine DNA glycosylase (TDG). Excision by TDG results in an abasic site that is processed in a replication‐independent manner by AP endonuclease and downstream base excision repair activities to produce an unmethylated cytosine (white). B, *Replication‐dependent DNA demethylation*: 5hmC is the most prevalent product of TET enzyme activity and is largely found at CpG sites. DNA containing fully methylated CpGs is converted to strands of hemi‐methylated DNA during replication (right side of panel). The DNMT1/UHRF complex maintains DNA methylation during replication by targeting hemi‐methylated DNA and methylating the newly incorporated, unmethylated cytosine (white) in the complementary strand to 5mC (black). 5hmC (orange), however, is not/poorly recognized by the DNMT1/UHRF complex, as indicated (left side of panel). The “hemi‐hydroxymethylated” DNA is either further oxidized by TET enzymes during the remaining cell cycle or “diluted” by subsequent rounds of replication. Thus, in the absence of TET enzyme activity, methylated DNA remains undiluted by replication. C, *DNA demethylation by deamination*: When methylated cytosine is deaminated (as opposed to oxidized in A and B), thymine (blue) is produced. The presence of thymine opposite guanine, the natural base partner of cytosine, creates a DNA mismatch. TET enzymes are also capable of oxidizing thymine, resulting in 5hmU (fuchsia). Both thymine and 5hmU, when mismatched with G, are specific substrates for TDG; mismatched thymine and 5hmU are also specific substrates of MBD4 and SMUG1, respectively

So far three mechanisms for demethylation have been identified—active demethylation (replication independent), passive demethylation mediated by TET (replication dependent), and 5mC deamination (Figure 1). Although far less established, and probably less prevalent than the two TET‐mediated pathways, evidence continues to grow suggesting the presence and importance of deamination in regulation of demethylation.

### 2.1 5‐Methylcytosine demethylation—replication independent

**FIGURE 2:**
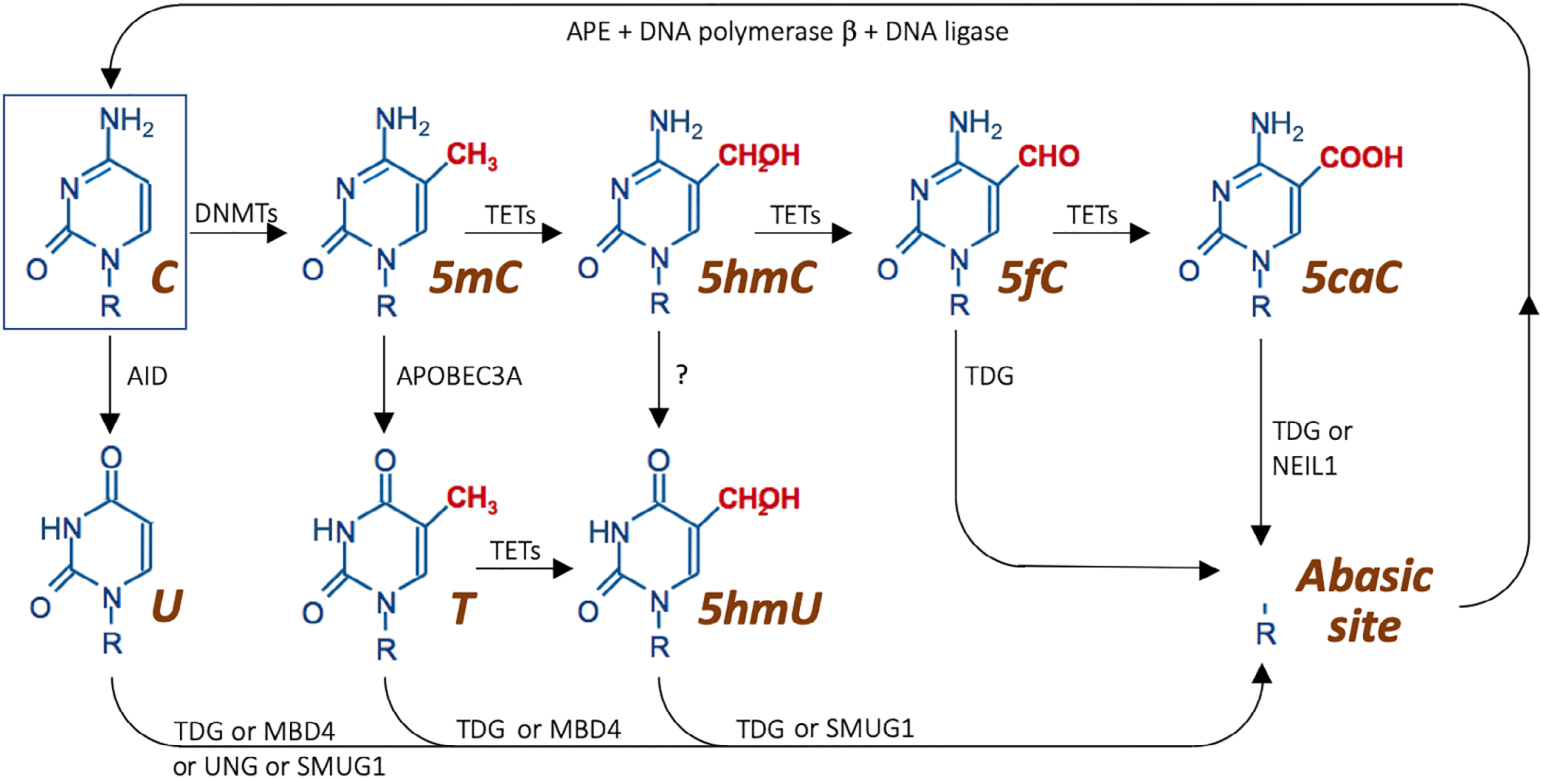
Biochemical details of pathways of DNA demethylation. Schematic of DNA demethylation pathways. 5caC, 5‐carboxylcytosine; 5fC, 5‐formylcytosine; 5hmC, 5‐hydroxymethylcytosine; 5hmU, 5‐hydroxymethyluracil; 5mC, 5‐methylcytosine; AID, activation‐induced deaminase; AP site, apurinic/apyrimidinic site; APE, AP endonuclease; APOBEC3A, apolipoprotein B RNA‐editing catalytic component 3A; C, cytosine; DNMTs, DNA methyltransferases; MBD4, methyl‐binding domain 4; NEIL1, Nei‐like 1 glycosylase; SMUG1, single‐strand selective monofunctional uracil DNA glycosylase 1; T, thymine; TDG, thymine DNA glycosylase; TETs, ten‐eleven translocation dioxygenases; U, uracil; UDG, uracil DNA glycosylase

In the “replication‐independent” active demethylation, TET dioxygenases sequentially oxidize 5mC to 5‐hydroxymethylcytosine (5hmC), and further to 5‐formylcytosine (5fC), and finally to 5‐carboxylcytosine (5caC).^8^, ^9^, ^10^ 5fC and 5caC are then removed by base excision repair (BER), namely by DNA glycosylases (Figure 2). As a result, the loss of methylated cytosine is not due to the lack of binding of the DNMT1‐UHRF in a replication‐coupled process (see below), but direct excision through BER. The main DNA glycosylase capable of excising the TET‐generated 5fC and 5caC is TDG^9^, ^10^, ^11^; in addition, Nei‐like 1 DNA glycosylase (NEIL1) can also remove 5caC, but not 5fC.^12^, ^13^ Of note, the partial redundancy of TDG and NEIL1 appears to be mechanistically relevant, as NEIL1 directly interacts and enhances TDG activity on 5caC.^13^ Upon removal of 5fC and 5caC, the resulting apurinic/apyrimidinic/abasic (AP site) is cleaved by AP endonuclease (APE); the one residue gap is filled by DNA polymerase β and finally the nick is sealed by DNA ligase.^14^

This pathway is very significant in development, likely due to the need for rapid programming of cellular state changes for pathway commitment, for example, myeloid vs lymphoid, neural vs neural crest. In another word, aggressive demethylation is needed since cells are rapidly proliferating but also malleable—a critical point). The clear evidence of the developmental significance of this pathway is the embryonic lethality phenotype of TDG null mice.^15^, ^16^

### 2.2 5‐Methylcytosine demethylation—replication dependent

Characterization of TET enzyme function and recruitment has shown that the most prevalent process of demethylation is linked with replication and mediated by gene expression and reprogramming via transcriptional states.^17^, ^18^, ^19^ The model is as follows: (a) frequent transcription of genes results in the recruitment of TET enzymes by transcription factors; (b) TET enzymes induce the oxidation of 5mC to 5hmC; (c) 5hmC bases are poorly recognized by the DNMT1‐UHRF complex, which targets and fully methylates the transiently hemimethylated strands during replication; (d) after oxidation, the strand becomes hemi‐methylated following one round of replication, and then fully demethylated after two rounds of replication. Thus, this mechanism of demethylation is likely a reflection of transcriptional activity, rather than induction of transcription per se.

This model is consistent with the fact that “CpG islands”—large intragenic CpG‐rich regions that act as cis‐elements at gene promoters—are largely unmethylated, that is, methylation is inversely proportional to gene expression. Accordingly, the role of TET enzymes in mediating passive demethylation is very significant during differentiation, when a lot of transcriptional activities occur. This is likely due to the need to maintain lineage and pathway commitment once established (ie, once the ball is rolling, keep the pace since the cells are proliferating quickly), as is the case for differentiation of immune progenitors to mature immune cells.^20^ One potential problem with this model is that DNMT1 activity is affected by the oxidation state of the hemi‐methylated strand—its activity is only about a third on the Cs opposite to 5hmCs compared to those Cs opposite to 5mC.^21^

### 2.3 Deamination of cytosine and 5‐methylcytosine

Deamination of cytosine is one of the most characterized processes of cytosine modification, but the least understood in terms of pathway involvement. While pyrimidines are known to be prone to spontaneous deamination,^22^, ^23^ enzymatic deamination of cytosine is driven by the AID/APOBEC family of deaminases, turning cytosine into uracil and resulting in the generation of a G:U mismatch. The resulting uracil in the G:U mismatch can be processed by any of four BER DNA glycosylases: uracil DNA glycosylase (UDG, encoded by *UNG*), TDG, methyl‐binding domain‐4 (MBD4), and single‐strand selective monofunctional uracil DNA glycosylase (SMUG). In addition to BER alone, cytosine deamination can be repaired in a concerted manner by both BER and mismatch repair (MMR) enzymes. The model proposed by Girelli Zubani et al describes two possible forms of G:U MMR—(a) DNA glycosylase‐mediated base excision and nick formation at the mismatch or (b) MLH1/PMS2‐mediated endonuclease activity away from the mismatch. In both cases, error prone repair is mediated by Polη and ExoI, ultimately leading to A/T mutagenesis. The authors convincingly argue that both processes contribute equally as the rate of A/T mutagenesis is only significantly decreased in the *Pms2*‐*Ung* double‐knockout mice, compared to respective single knockout.^24^ This “error‐prone” system is utilized in a controlled fashion to diversify the antibody affinity during immunoglobulin switch and somatic hypermutation (SHM), and the nontrivial role of DNA glycosylase repair may allude to the involvement of DNA methylation.^24^

Deamination has an inherently important role in the context of DNA methylation. In fact, deamination of 5mC produces thymine, generating a G:T mismatch in the CpG context. Only two enzymes are known to excise thymine in this unique context, TDG and MBD4. While TDG is known for its causing significant embryonic lethality phenotype when mutated, and its defined role in active demethylation,^15^, ^16^ *Mbd4* mutant mice develop normally and do not show a dramatic increase in cancer susceptibility^25^, ^26^, ^27^; however, an increased number of C to T transition mutations at CpG sites are noted, that is, CpG to CpA or CpG to TpG, as expected due to unrepaired G:T mismatches.^25^, ^26^ In addition, when *Mbd4* mutant mice were crossed into mice mutant for the tumor suppressor gene *Apc* or the MMR gene *Mlh1*, an increased tumor burden and rate of tumor progression is noted.^25^, ^26^, ^28^ This suggests that MBD4 may serve as a possible link between deamination and demethylation.

## 3 LINKS BETWEEN DEMETHYLATION AND DEAMINATION

Recently, a demethylation pathway involving TET1, AID, and MBD4 was uncovered in mouse embryonic cells; the loss of function of any of these genes resulted in hypermethylation of the transfected CpG island reporter, but not endogenous CpG islands.^29^ The model suggested by the authors is that in contrast to maintenance of the overall bimodal methylation pattern, a specific pathway can be activated for “resetting” methylation patterns during somatic cell reprogramming. It is interesting that this putative pathway might be at the intersection between the TET‐mediated oxidation and AID/APOBEC‐mediated deamination of methylated CpGs.

Indeed, previous studies have correlated AID with DNA demethylation. For example, our group has shown in vivo the direct interactions between AID and TDG,^16^ but has not functionally tested the involvement of AID or other deaminases in demethylation. MBD4 was shown to be involved in active DNA demethylation in zebrafish, acting in concert with AID and the adaptor protein GADD45,^30^ but some of these data is considered controversial.^31^ However, since many of these studies were conducted prior to the discovery of TET enzyme function and their establishment as prominent mediators of active demethylation, the functional significance of these interactions needs to be reexamined.

Most work investigating AID/APOBEC enzymes in the context of CpG methylation has focused on characterizing the substrate specificities for 5mC and TET‐oxidized derivatives. The results of in vitro biochemical studies demonstrated that most AID/APOBEC family enzymes have physiologically insignificant affinities for 5mC, largely dismissing the concept of demethylation through deamination.^32^ However, studies over the past 5 years reintroduced the concept of deamination as affecting cytosine methylation, through either a direct or indirect biochemical process. APOBEC3A was recently shown as being capable of significantly deaminating 5mC, providing a direct molecular mechanism of generating T:G mismatches from 5mC.^33^ Sabag et al, as mentioned previously, showed that the maintenance of the demethylated state in CpG islands in embryonic stem cells is disrupted in TET1 knockout cells, as expected, as well as in AID, MBD4, and GADD45a null lines.^29^ As further studies showed the interactions of AID and GADD45a with TDG,^16^ a link between demethylation and deamination has been reinforced.

A recent study further advanced the role of GADD45a in demethylation, and possibly deamination, by demonstrating a mechanistic link with TET1. The prevailing paradigm for TET enzyme targeting for demethylation is through its recruitment by transcription factors to transcriptionally active regions. However, details beyond protein‐protein interactions have been largely unknown. Recent work by Niehrs and collegues makes a significant progress in identifying a process of TET1 recruitment to CpG islands in promoters through DNA‐RNA hybrids, or R‐loops.^34^ Through antisense transcription of the region, a long noncoding RNA (lncRNA) is produced resulting in R‐loop formation that is then bound by GADD45a, which subsequently recruits TET1 and induces demethylation. Whether this process is complementary or parallel to the role of transcription factor‐induced TET recruitment is an important question that may point to a role for deamination.

Arguably the most significant limitation in the initially proposed deamination‐demethylation pathways was the assumption that oxidized uracil compounds, 5‐hydroxymethyluracil (5hmU), 5‐formyluracil, and 5‐carboxymethyluracil, which are considered to be putative substrates for TDG, MBD4 and SMUG, were derived from deamination of the TET‐oxidized 5mC derivatives. However, a functional study investigating 5hmU showed that the very TET enzymes could oxidize *thymine* to produce 5hmU physiologically.^35^

In vivo studies support the presence of a deamination‐mediated demethylation pathway, possibly intersecting with TET enzyme function. Guo et al demonstrated that overexpression of TET1 in HEK293 cells increased 5hmC, as expected. Intriguingly, the increase in 5hmC was reduced when the deaminase AID was co‐overexpressed with TET1.^36^ To investigate whether deaminases were involved in 5hmC demethylation, the authors transfected HEK293 cells with PCR products containing 5hmC. Using bisulfite‐sequencing, a genomic DNA methylation analysis method, they found that, when co‐transfected with AID or APOBEC family expression constructs, the PCR products showed increased rates of demethylation. The sequence context of 5hmC demethylation showed a 2.4‐fold higher rate of demethylated residues in the WRC vs SYC context (where W is A or T, R is A or G, S is G or C and Y is C or T—IUPAC nucleotide codes), reflective of the AID sequence selectivity for WRC motifs. Finally, overall distribution of the 5hmC demethylation deviated significantly from the expected Poisson distribution, suggestive of a processive (ie, by scanning), and not distributive (ie, by random targeting), mechanism of demethylation.^36^

The concept of deamination and processive demethylation has since been investigated, but remains significantly understudied overall. Franchini et al used a DNA targeting domain (GAL4) fused to AID and a *Xenopus* extract system to show that deamination in the vicinity of 5mC residues induced demethylation.^37^ The group then applied the same targeting approach in vivo, breeding transgenic GAL4‐AID mice to transgenic mice bearing the GAL4 binding sites (UAS) into the first of four paternal (methylated) *H19* differentially methylated regions (DMRs). The results showed demethylation throughout the region, irrespective of GAL4 binding motif context, that is, not just of the first DMR, making AID induced deamination of each CpG very unlikely and instead suggesting involvement of a processive DNA repair pathway (ie, long patch BER or MMR).^37^

**FIGURE 3:**
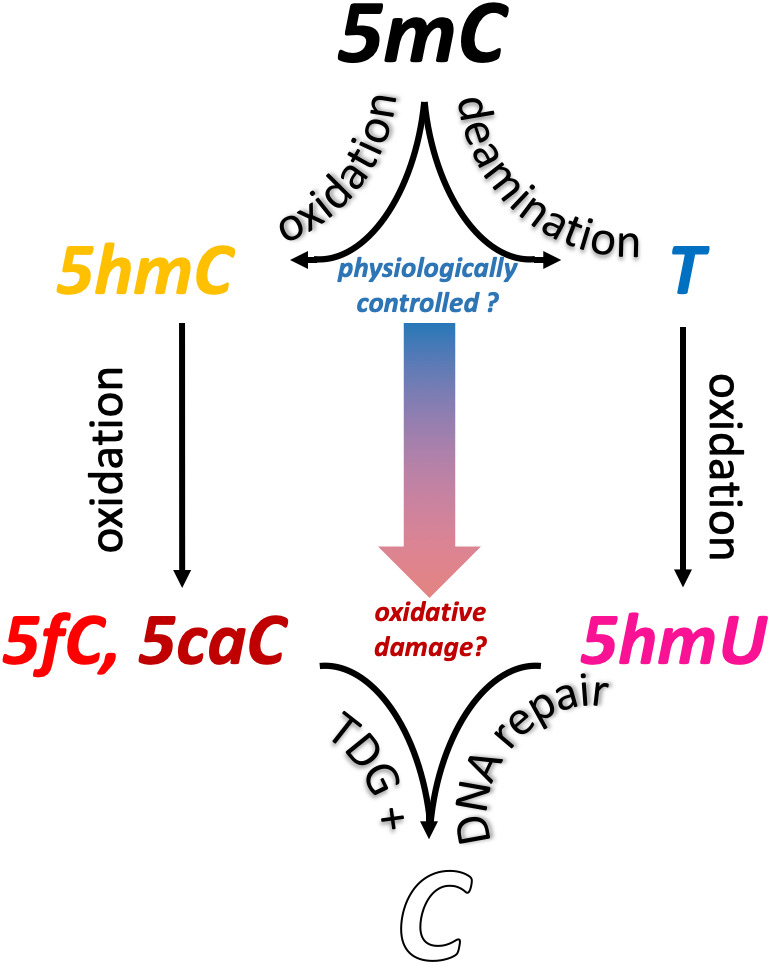
Blurred boundaries between physiological DNA modifications and pathological DNA damage: the underlying central role of thymine DNA glycosylase (TDG) and base excision repair in active demethylation. The production of stable 5hmC oxidation derivatives (5fC and 5caC) by ten‐eleven translocation dioxygenase (TET) enzymes has been well established in vivo but their function remains unresolved. Conversely, 5hmU has long thought to be a form of oxidative DNA damage, yet the regulation of deaminating enzymes is understudied, despite evidence of significant effects on the global DNA methylation state. Enzymatic DNA oxidation and deamination are conceptually linked through the production of substrates specifically excised by TDG. 5hmU, 5‐hydroxymethyluracil; 5mC, 5‐methylcytosine; 5hmC,5‐hydroxymethylcytosine; 5fC, 5‐formylcytosine; 5caC, 5‐carboxylcytosine; T, thymine. DNA bases are color coded as in Figure 1

While the functional relations between deamination and demethylation are intriguing, how the respective reactions might co‐exist is an open question. Why do enzymes such as SMUG1 and TDG act on 5hmU and related residues, if it is unlikely that AID/APOBEC deaminases generate them in the first place? If TDG, an evolutionarily conserved enzyme with an embryonic lethal knock‐out phenotype, has a primary role in demethylation, then why does it have overlapping roles in excising both 5hmC oxidation products (5fC, 5caC) and potential deamination products (T, 5hmU)? One simple yet uninvestigated possibility is that the oxidation reactions by TET enzymes may exacerbate the effects of aberrant deamination. If aberrant deamination of 5mC occurs (ie, by APOBEC3A, thereby producing thymine) in regions of high TET activity, then 5hmU becomes an additional source of DNA damage. In short, AID/APOBEC enzymes may increase the need for a “guardian” of TET (over‐)activity; with this perspective, TDG might have a well‐designed role in maintaining the genomic code (Figure 3).

The most striking example of the relationship between (de‐)methylation and deamination is in their effects on genomic CpG mutagenesis. While the concept of demethylation driven solely by deamination has clearly not been established, the likely intersection between TET and AID/APOBEC enzyme function can clearly be seen in their mutagenic patterns (Table 1). Further studies will reveal whether TET‐mediated oxidation and AID/APOBEC‐mediated deamination function together as a synchronized pathway or independently to induce controlled CpG mutagenesis.

**TABLE 1:** TET and AID/APOBEC mutational effects at CpG sites

Context	SNV, motif	Trend and genomic bias	Experimental system	References
5hmC	C > T	Decreased at CpG sites	Human	38
5hmC	C > G	Increased at 5hmC sites	Mouse, human	39
TET1 KO	C > T	Increased at CpG sites in coding genome	Mouse, cancer	40
TET2 KO	C > T, WRC motif	Increased in coding genome	Mouse, cancer	41
TET2 KO	C > T	Increased at CpG sites with 5hmC within coding genome	Mouse, cancer	42
TET2/3 DKO	C > T	increased at CpG sites, euchromatin	Mouse, cancer	43
TET2/3 DKO	C > T	Increased bias at V_H_186.2	Mouse, cancer	44
APOBEC3A activity	C > T, YTCR motif	Within DNA hairpins; putative cancer driver hotspots	In vitro, human, cancer	45
High AICDA expression	C > T, WRCG motif	Increased at highly methylated CpG sites Negative correlation mutation vs methylation	Human, cancer—lymphoma	46
High APOBEC expression	C > T	Increased at CpG sites, unmethylated Negative correlation methylation vs mutation	Human, cancer—TCGA	47
High APOBEC3B expression	C > T	Increased at CpG sites	Human, cancer—TCGA	48
High APOBEC3 expression	C > T, TCW motif	Increased at non‐Ig loci no correlation to CpG sites	Human, cancer—CLL	49

A significant element of complexity in uncovering the overlap between methylation and deamination in the genomic context is the reliance on the bisulfite sequencing method, currently the gold standard for identifying methylated cytosines.^50^, ^51^ Bisulfite sequence analysis identifies methylated cytosines by deaminating all unmethylated but not methylated cytosines. However, as a result of this in vitro deamination, any cell‐produced uracil is indistinguishable from bisulfite‐generated uracil. One approach to address this problem is to compare untreated to bisulfite‐treated sequences with single‐base resolution in “normal” vs AID/APOBEC contexts, allowing a complete assessment of the cytosine modification state.

## 4 THE SIGNIFICANCE OF 5mC OXIDATION DERIVATIVES

The biochemical pathway of sequential oxidation of 5mC clearly points to TDG as the terminal enzyme, due to its specific excision of 5fC and 5caC.^9^, ^10^, ^11^ The presence of this system is reflected in vivo*—*overexpression of TET enzymes increases 5fC and 5caC, while loss of TET activity leads to the reduction of 5hmCas well as 5fC and 5caC.^8^, ^9^, ^10^, ^52^ Accordingly, loss of TDG leads to an *increase* in 5fC and 5caC, in support of its function as the primary excision enzyme of these substrates.

Shortly after being identified as the terminal enzyme in 5mC oxidation, the possibility of TDG affecting DNA methylation was investigated by us as well as by Schär's group. Using knockout strategies, both groups identified TDG as being essential for embryonic development in mice, and showed that DNA methylation increases at developmentally relevant promoter and enhancer sites.^15^, ^16^ In addition, our group also described the demethylation defect of homozygous Tdg knock‐in mice bearing a point mutation (*Tdg* ^*N151A*^) that inactivated its glycosylase activity, thus providing compelling genetic evidence on the existence of an active, enzymatically driven DNA demethylation process.^16^, ^53^ Furthermore, our recent work shows evidence of increased de novo methylation following TDG knockdown in melanoma cell lines^54^ and mouse adenomas bearing the *Tdg* ^*N151A*^ knock‐in mutation,^55^ respectively. In summary, the studies using three genetic systems across two mammalian species strongly support the role of TDG in preventing aberrant DNA methylation in the form of elevated 5mC.

The mechanism of how TDG affects DNA methylation physiologically is an important but unanswered question. The simplest explanation is that the accumulation of 5fC and 5caC results in negative feedback producing 5mC. In this scenario, an increase in global 5fC and 5caC would precede a global increase in 5mC. While the first part of this possibility has been consistently demonstrated, the kinetics of 5mC accumulation has not, and thus warrants more in‐depth investigation. Additional possibilities are more complex and imply a direct effect of 5fC and 5caC. One study demonstrated that 5caC accumulation in TDG null mouse embryo fibroblasts induces ectopic CTCF binding sites across the genome.^56^ CTCF is a multifunctional transcription factor that binds to DNA in a sequence specific manner influenced by CpG methylation, and has insulator activity, blocking enhancer‐promoter communications. The best characterized example of its function is in imprinting regulation of IGF‐2 expression: when the CTCF binding site is demethylated, CTCF binds and induces a methylation change *downstream*, thereby repressing IGF‐2 expression. Thus, the presence of ectopic CTCF sites can be predicted as having dramatic changes in methylation patterns that need not be reflected as a global increase in 5mC.

5fC and 5caC may also serve as functional epigenetic marks with effects beyond 5mC and 5hmC levels. Perhaps the most compelling evidence points to a function in chromatin. 5fC can form DNA protein crosslinks with nucleosomes.^57^, ^58^ Similar to CTCF, ectopic nucleosomes could induce reciprocal change in DNA methylation patterns without a global increase. Thus, the possibility that 5fC and 5caC induce ectopic repressive marks at a chromatin level, which then result in focal, but not global, DNA methylation increases, is an intriguing and important unanswered question. Raiber et al showed that 5fC enhanced nucleosome occupancy through stabilization via lysine‐Schiff base crosslinking.^59^ This increased occupancy occurs at active enhancers, correlating with increased expression of associated genes, in a tissue specific manner.^59^ Another recent study showed reduced TDG enzyme activity at nucleosome bound 5fC that contained histones H2A.Z and H3.3, two variants enriched at enhancer sequences.^60^ Interestingly, incubation with transcription factor FOXA1 caused increased TDG activity at 5fC sites, consistent with a reported ability of FOXA1 to open compacted chromatin.^60^, ^61^ Examples of transcription factors preferentially binding to 5caC containing recognition sites have been reported, possibly functioning to promote open chromatin in a manner similar to FOXA1.^62^, ^63^, ^64^, ^65^

Perhaps the strongest evidence for 5fC and 5caC oxidation derivatives functioning as markers of active chromatin is in the context of p300, an essential histone acetyltransferase. Song et al used two methods of 5fC genome‐wide profiling to show the enrichment at enhancers as well as a correlation with acquired sites containing p300.^66^ As the authors note, this may reflect active 5mC oxidation to facilitate p300 binding. In line with this concept, studies have shown TDG to enhance CBP/p300 mediated transcription through allosteric interactions.^67^, ^68^, ^69^ Similarly, absence of TDG in vivo results in a reduction of histone acetylation, with in vitro studies confirming TDG as a co‐activator of p300 activity.^70^

However, the possibility that 5fC and 5caC are inevitable oxidation byproducts of TET enzyme activity cannot be ruled out. Evidence for this lies in characteristics of these bases resembling oxidative damage.^71^, ^72^, ^73^, ^74^, ^75^, ^76^, ^77^, ^78^, ^79^ A mutagenic effect of 5fC was demonstrated prior to the discovery of the TET enzyme mechanism of formation, whereas 5caC was also shown to induce MMR protein activity.^72^, ^80^ Furthermore, both 5fC and 5caC induce a reduction in transcriptional efficiency—a counterintuitive effect considering the link between TET enzyme mediated demethylation and transcriptional *activation*.^57^, ^81^ Thus, transcriptional regulation by DNA (de)methylation/deamination should be considered, in all respects, a repurposing of DNA damage during evolution, by which chemical “lesions” have been redeployed as physiological elements of the epigenetic syntax, which again required the involvement of DNA repair enzymes like TDG to “check and balance” dioxygenase/deaminase activity in order to maintain genomic and epigenomic stability (Figure 3).

**FIGURE 4:**
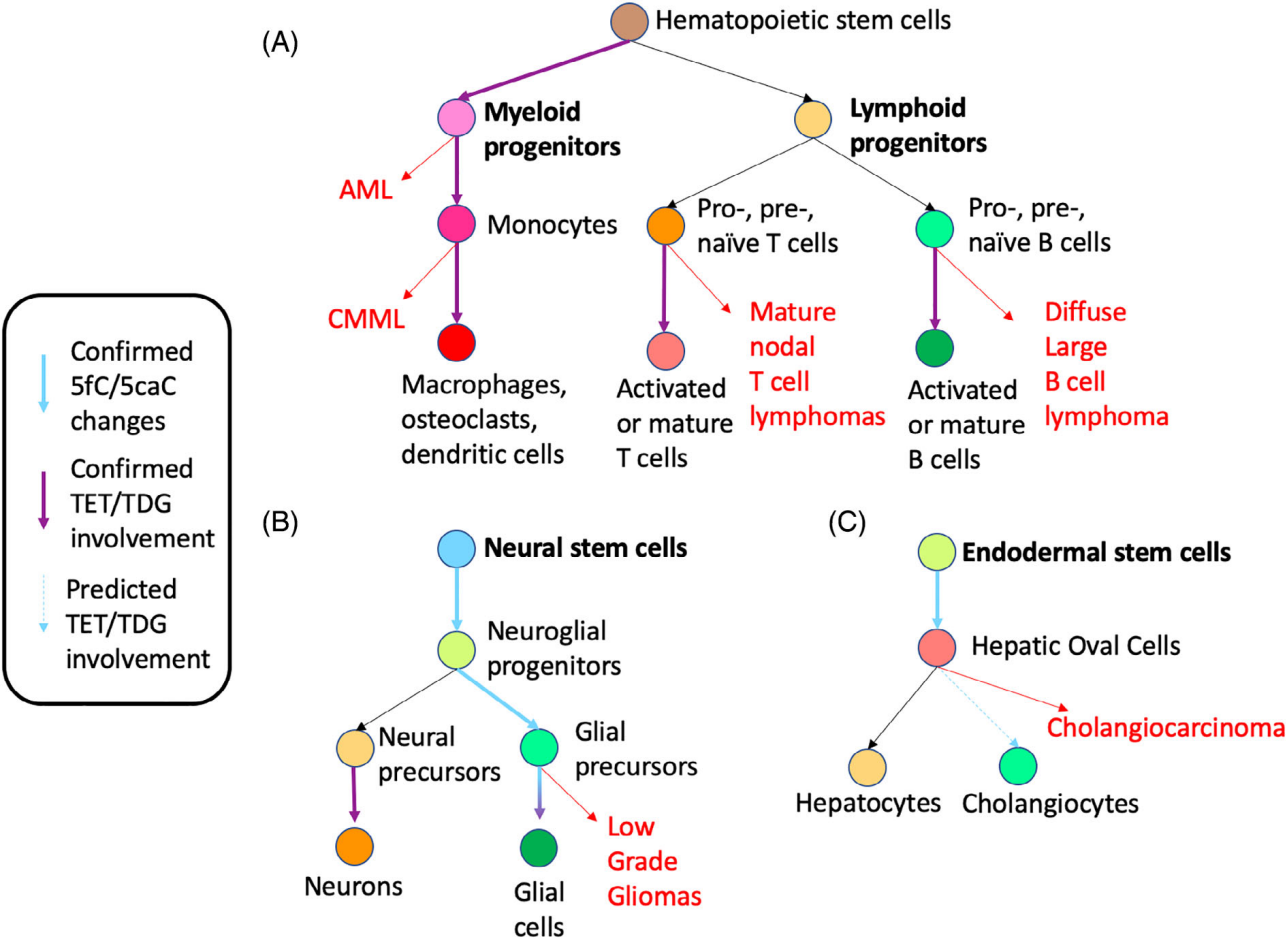
(De)methylation underscores the transition points between oncogenesis and differentiation. The developmental progression of hematopoietic, neural, and hepatic cell lineages is summarized and depicted in “rolling marble” format. Colors of lineages annotated in Table 2 are conserved and bolded. Associated cancers from Table 2 are in red. Solid blue arrows represent transitions reported to involve changes in 5fC/5caC levels.^82^, ^83^, ^84^, ^131^ Solid purple arrows represent transitions reported to involve ten‐eleven translocation dioxygenase (TET)/thymine DNA glycosylase (TDG) enzymes, discussed in text. Dashed blue arrows represent transitions predicted to involve changes in 5fC/5caC levels

While the question of 5fC and 5caC as functional epigenetic marks vs inevitable byproducts remains equivocal, demethylation overall plays a vital role in mammalian development that continues to be delineated. In the context of organogenesis, much progress has been made in neural development, the system most enriched for 5mC oxidation derivatives. Transient changes in 5fC/5caC have been observed in neural lineage specification and in hepatic organogenesis^82^, ^83^, ^84^ (Figure 4). In the hematopoietic system, TET KO mouse studies continue to demonstrate the complex but important roles in the immune system development and maturation, as discussed in a recent review^132^ and below in this article.

## 5 THE CLINICAL SIGNIFICANCE OF DNA (DE)METHYLATION REFLECTS INVOLVEMENT IN DEVELOPMENT AND CANCER

The spectrum of clinically significant mutations bridges the developmental functions of (de)methylating enzymes with their roles in oncogenesis. To illustrate this point, the incidences of the most commonly mutated DNA (de)methylating enzymes (DNMT3A, TET2, and isocitrate dehydrogenase [IDH] 1/2) are listed with the associated cancers, and graphically represented in the context of developmental lineages (Table 2 and Figure 4). IDH 1 and 2 mutations are included as a presumed physiological equivalent to reduced activities of all three TET enzymes. This assumption is based on the roles of IDH1 and IDH2 in the production of alpha‐ketoglutarate, an essential co‐factor for TET‐mediated 5mC oxidation, and the observation that the most frequent *IDH1*/*2* mutations result in the production of 2‐hydroxyglutarate, a global TET enzyme inhibitor. Accordingly, these mutations result in a hypermethylation phenotype and loss of 5hmC, and are mutually exclusive with *TET2* mutations in AML.^133^

**TABLE 2:** Pooled prevalence of *DNMT3a*, *TET2*, and *IDH1/2* mutations in specific cancers

		*DNMT3a*			*TET2*			*IDH1/2*			
Lineage	Cancer type	Mutated (or cancer present)	Total	Prevalence^a^ (%)	Mutated (or cancer present)	Total	Prevalence^a^ (%)	Mutated (or cancer present)	Total	Prevalence^a^ (%)	References
Myeloid	Primary AML	197	840	**23.5**	172	945	**18.2**	42	208	**20.2**	85, 86, 87, 88, 89, 90, 91
	CMML	22	367	**6.0**	247	592	**41.7**	4	165	**2.4**	85, 92, 93, 94, 95, 96, 97, 98, 99, 100
Lymphoid	Mature, nodal T‐cell lymphomas	113	407	**27.7**	281	551	**50.9**	45	226	**19.9**	101, 102, 103, 104, 105, 106, 107, 108
	Diffuse large B‐cell lymphoma	9	1711	**0.5**	119	2338	**5.1**	0	1049	**0**	101, 109, 110, 111, 112, 113, 114, 115, 116, 117, 118
Neural	Low grade glioma	4	512	**0.8**	2	512	**0.4**	418	512	**81.6**	119
	GBM	4	397	**1.0**	0	397	**0**	25	397	**6.3**	120
Endoderm	HCC	4	366	**1.1**	1	366	**0.3**	6	366	**1.6**	121
	Cholangiocarcinoma	0	36	**0**	0	36	**0**	16	98	**16.3**	122, 123
Germline	Primary AML	3	16	**18.8**	1	14	**7.1**	2	56	**3.6**	124, 125, 126
	CMML	—		—	1	14	**7.1**	—		—	127, 128
	Lymphoma—T‐cell origin^b^	—		—	2	14	**14.3**	—		—	129, 130
	Lymphoma—B‐cell origin^c^	—		—	5	14	**35.7**	—		—	
	Low grade glioma	—		—	—		—	6	56	**10.7**	
	GBM	—		—	—		—	0	56	**0**	
	HCC	—		—	—		—	0	56	**0**	
	Cholangiocarcinoma	—		—	—		—	2	56	**3.6**	

Abbreviations: AML, acute myeloid leukemia; CMML, chronic myelomonocytic leukemia; DLBCL, diffuse large B‐cell lymphoma; GBM, glioblastoma multiforme; HCC, hepatocellular carcinoma; LGG, low grade glioma; NLPHL, nodular lymphocyte predominant Hodgkin lymphoma.

^a^ The number of individuals with somatic mutation were pooled from indicated references. Multiple mutations in the same individual were counted as a single case. Mutations in both genes were not included in this analysis (with the exception of IDH2 and TET2 in AITCL).^108^ For germline mutations, prevalence reflects number of reported cancer cases among individuals with germline mutation.

^b^ Lymphoma of T‐cell origin (germline)—T‐cell origin: T‐cell lymphoma; nodal peripheral T‐cell lymphoma of T follicular helper.

^c^ Lymphoma of B‐cell origin (germline)—B‐cell origin: NLPHL; EBV‐positive Hodgkin‐like polymorphic B‐cell lymphoproliferative disorder; primary mediastinal large B‐cell lymphoma.

When analyzing the cumulative incidences (Table 2), few notable observations can be made. The incidence of mutations is by far most significant in low‐grade glioma, consistent with the brain having the highest enrichment of 5mC oxidation derivatives. *TET2* mutations are more frequent in “mature” type hematopoietic cancers, consistent with its defined function in terminal differentiation/maturation within both myeloid and lymphoid lineages.^132^ The incidence patterns of specific cancers in patients carrying germline mutations appear to resemble the somatic mutation patterns, likely in further support of respective developmental functions. One notable exception is the high incidence of *TET2* somatic mutations in T‐cell lymphomas vs B‐cell lymphomas in germline mutation carriers, which may reflect differences in survival observed in *Tet2* KO mouse models.^134^ Finally, the abundance of *IDH1/2* mutations in gliomas and cholangiocarcinomas, but lack of *TET2* mutations, are likely a reflection of TET enzymes functioning in tissue specific patterns—TET1, for example, has very recently been shown to be an oncogenic driver in IDH‐wt cholangiocarcinoma.^135^

## 6 CYTOSINE MODIFICATION AND IMMUNE FUNCTION

### 6.1 Methylation

The concept of dynamic epigenetic marks has been investigated in the hematopoietic system, with mapping of DMRs showing specific signatures in the differentiation of progenitor cells.^136^ In addition to direct influence on gene expression (ie, methylation of promoter regions), methylation changes in specific transcription factor binding sites seem to maintain predefined “differentiation trajectories.” A clear example is in the commitment of progenitor cells to lymphoid vs myeloid differentiation, in which lymphoid precursors acquire global increase in methylation at DMRs throughout the genome.^136^ Notably, this paradigm extends into lymphoid cell maturation: a whole genome analysis of B‐cell differentiation showed a progressive accumulation of methylation changes in each progenitor stage, affecting 30% of all autosomal CpG sites.^137^

While the effects on gene expression are variable, the role of methylation is reinforced in physiological models: in mice with DNMT1 knockdown and subsequent CpG hypomethylation, myeloid cell development, and numbers are normal, while lymphoid development is deficient. Interestingly, in the same study, Broske et al noted that cell numbers of all hematopoietic stem cell (HSC) derivatives were normal in mice lacking de novo DNMTs DNMT3A and DNMT3B. This suggests that additional DNA methylation is dispensable in defining the fate of stem cells and further highlighting the nuanced but significant differences within methylation‐dependent effects.^138^ In parallel to the global methylation changes noted with B‐cell differentiation, however, a direct functional role for methylation in immune function is apparent at the IgK light chain locus. Here the methylation status of these sites appears to be regulated by DNMT3A and DNMT3B, as conditional knockout in mice produces precocious Vk‐Jk recombination in B‐cell progenitor cells.^139^

Finally, the significant role of DNMT enzymes in HSC development is clinically apparent in Immunodeficiency, centromeric instability and facial anomalies syndrome, a rare but notable developmental disease caused by loss‐of‐function mutations in DNMT3B. The clinical hallmark of this syndrome is agammaglobulinemia causing immunodeficiency, which most often leads to early age fatalities. Although patients exhibit levels of lymphoid cells in peripheral blood ranging from normal to low, the significant impact of this disease on immune function highlights the nuanced but potent relationship between methylation and hematopoietic system.^140^

### 6.2 DNA (de)methylation and immune function

The significance of both methylation and demethylation in differentiation is not limited to embryonic development or lineage commitment—it is an essential and continual process in the immune system. As in HSC progression, the combination of noncoding factors (eg, lncRNAs) and transcription factors is linked with DNA methylation status, and as such, the differentiation and maturation of both erythroid and lymphoid cells are a model for the roles of all three pathways of DNA demethylation.

As mentioned previously, TET enzymes are primarily recruited to enhancer regions by transcription factors. This is consistent with the current paradigm that the majority of DNA demethylation is induced by transcriptional activation as a positive feedback to enhance chromatin accessibility.^141^, ^142^, ^143^, ^144^, ^145^, ^146^, ^147^ Demethylation coupled to transcription factor binding sites is also seen in plasma cell differentiation, as well as in every type of thymocyte: differentiation of naïve CD4 T cells, memory cell differentiation in CD8 T cells, lineage modulation of invariant natural killer T cells, and Foxp3‐expressing T regulatory (*T*_reg_) cells.^148^, ^149^, ^150^ Another example of TET functions is in IgK rearrangement and B‐cell differentiation, wherein, as mentioned above, the loss of de novo DNMTs leads to precocious rearrangement.^139^ Accordingly, the TET proteins have been shown to regulate IgK rearrangement by oxidizing 5mC at enhancer regions.^144^ For more detail, the reader is referred to a recent review describing the role of TET enzymes and passive demethylation in immune maturation and function.^132^

The strongest example of active demethylation involvement in the hematopoietic system is in a recent study by Suzuki et al investigating the master transcription factor RUNX1. Overexpression studies showed the binding site‐directed DNA demethylation, and co‐immunoprecipitation demonstrated the interactions between TET enzymes and TDG. Furthermore, analysis in noncycling cells also showed the RUNX1‐mediated DNA demethylation, serving as convincing support of active demethylation as opposed to a passive process depending on replication.^151^

### 6.3 Deamination

Cytosine mutagenesis mediated by deamination plays an important role in SHM of immunoglobulin genes. Uracil residues introduced from cytosine deamination by AID or APOBEC are processed by uracil glycosylases and repaired by MMR enzymes. Counterintuitively, the critical nicking complex of PMS2/MLH1 has been shown to be dispensable for the generation of mutations at A:T base pairs. However, a recent study showed that the PMS2/MLH1 pathway of mutagenesis acts in concert with uracil glycosylases in strand‐specific manners that expand the overall mutation spectrum.^24^

In addition to SHM, DNA deamination plays a role in class switch recombination (CSR) as well. Similar to SHM, CSR relies on the generation of a DNA nick induced by uracil glycosylase/BER recognition and removal of uracil. In a study using B cells derived from hyper IgM patients with UDG mutations, the strand specificity of uracil excision from ssDNA was demonstrated by the lack of compensation by SMUG1. Thus, despite a mechanistic overlap between DNA glycosylases, their in vivo functions appear to be more specific and regulated, as evidenced by strand specificity.^152^ Similarly, MBD4 interacts with MMR to facilitate CSR.^153^, ^154^

Notably, a less publicized but physiologically critical role for AID and deamination is in the suppression of autoreactivity. As AID was originally identified as the driver of antibody diversification through the previously mentioned processes (ie, SHM, CSR), expression and function of AID was thought to be limited to activated and mature B cells. Interestingly, low level of AID expression in immature B‐cells in human bone marrow is essential for suppressing autoantibody development through induction of apoptosis.^155^, ^156^ The consequence of increased autoreactive B‐cell clones following AID knockdown may very well allude to a phenotype‐modifying role in lymphoma; indeed, autoimmune paraneoplastic syndromes are a common yet highly variable occurrence in lymphomas.^157^

Although the mechanisms of demethylation through deamination remain unresolved, recent studies continue to demonstrate their functional connections. AID expression has recently been shown to be directly regulated by the TET enzymes.^158^ Recent work by Levine and colleagues argues that AID is an epigenetic regulator of myeloid/erythroid differentiation, through DNA methylation changes in HSC. Kunimoto et al showed that both TET2 and AID impact HSC differentiation through altered transcription via methylation changes at key promoters. Loss of function in both cases causes myeloid expansion and increased erythroid output. The roles of AID and TET2 do not seem to be redundant, however, as alterations in HSC self‐renewal and susceptibility to malignant transformation are seen with the loss of TET2, but not AID.^159^

## 7 MECHANISTIC ROLE OF DNA (DE)METHYLATION IN CANCER

### 7.1 Ten‐eleven translocations

The connection between demethylation and cancer has been strong from the beginning, with the TET enzymes being the first of many links. TET1, the first identified TET dioxygenase enzyme, was discovered in the early 2000s as the fusion partner to MLL in two cases of AML containing *t*(10, 11) translocations, one being in an 8‐year old patient.^160^, ^161^ Discovery of additional family members, TET2 and TET3, has solidified this link, with TET2 being the most clinically defined.

Somatic TET2 mutations were first reported as present in ~15% of myeloid cancers, with the loss of function mutations in TET2 accounting for approximately 10% of newly onset AML, 50% of CMML, and approximately one‐third of all mutations in myelodysplastic syndromes.^92^, ^93^ Clinically, TET2 mutations appear to have an unfavorable impact on prognosis of AML. In a most recent meta‐analysis by Wang et al, TET2 mutations predicted inferior overall survival as well as event‐free survival for patients with AML, and specifically in patients under 65 years of age (the authors noted that a data deficiency prevented an analysis of patients over 65 years of age) and those cytogenetically normal and intermediate‐risk AML.^162^ In the context of MDS, TET2 mutations do not appear to carry a significant prognostic value.^163^ Two independent meta‐analysis by Guo et al and Lin et al showed an incidence of 18.34% and 19.19%, respectively. In both studies, no significant difference in overall survival of the pooled cohorts was observed when comparing the patients with TET2 mutations to those without them.^94^, ^164^

The role of TET2 as a tumor suppressor is not limited to myeloid derived malignancies. TET2 mutations are present in an estimated 56% of patients with angioimmunoblastic T‐cell lymphoma and 46% of peripheral T‐cell lymphomas, suggesting that the TET2 loss has a more dramatic malignant phenotype in the lymphoid derivatives compared to the myeloid line.^101^, ^102^, ^103^, ^104^, ^105^, ^165^, ^166^ Notably, TET2 mutations are far less prevalent in diffuse B‐cell lymphomas at ~7% of reported cases.^109^, ^167^

The clonal expansion of leukemic‐driver mutations in clinically healthy patients has been defined as “clonal hematopoiesis of indeterminate potential” (CHIP), which was first proposed by Steensma et al.^168^ In line with their developmental and oncogenic significance, TET2 and DNMT3a mutations represent 93% of driver mutations in clonal hematopoiesis.^169^ Underscoring the systemic influence of the hematopoietic system, the significance of CHIP has been demonstrated not as an oncogenic precursor, but instead as an accelerator of atherosclerosis and chronic heart failure.^170^, ^171^, ^172^

The mechanism by which the TET2 loss‐of‐function mutations induce clonal expansion remains in question and likely holds the answer to an apparent paradox: despite the clear association between TET2 mutations and hyperproliferative states, TET2 mutations are not always identified as the driving or founding mutation in AML. The order of mutation acquisition has been investigated in one study in myeloproliferative neoplasms, where a severe clinical phenotype of polycythemia vera was observed when JAK2 mutations *preceded* TET2 mutations. Notably, the “TET2‐first” mutation acquisition appeared to dampen the upregulating gene expression effects of the pathogenic JAK2 mutation in a cell‐type specific manner.^173^ Further details regarding clonal hematopoiesis are discussed in cogent reviews by Bowman et al and Steensma.^168^, ^174^

While TET2 appears to have a clear role as a tumor suppressor, the function of TET1 in oncogenesis is equivocal as a tumor suppressor vs a driving oncogene. As mentioned above, the significance of TET1 was first noted as a fusion partner with the MLL gene in AML with the *t*(10, 11) translocation.^160^, ^161^ A subsequent study showed that TET1 was essential to the oncogenesis resulting from this fusion, primarily through regulation of the TET1‐MLL fusion protein with its significant target genes.^175^ The upregulation of TET1 coinciding with a mechanistic role is strongly supported by the global increase in 5hmC levels within these cells.^176^ In the case of solid tumors, a recent bioinformatic study reported elevated expression of TET1 in a subset of triple negative breast cancer cases in the Cancer Genome Atlas (TCGA; in comparison to normal breast tissue and hormone‐dependent tumors), which coincided with reduced methylation levels; in addition, TNBC cases with elevated TET1 expression levels and reduced methylation had worse prognosis.^177^ As mentioned previously, TET1 has also been shown to be an oncogenic driver in IDH‐wild‐type cholangiocarcinoma.^135^

In lymphoid lineage malignancy, however, TET1 appears to play a tumor suppressor role comparable to TET2. TET1 deletion in mouse models, both in isolation and in combination with TET2 deletion, produces B‐cell lymphomas.^40^, ^178^ Notably, TET1 as a tumor suppressor of B‐cell malignancy has also been supported by evidence of reduced TET1 expression in human non‐Hodgkin's lymphomas.^40^

### 7.2 AID and APOBEC3A

As discussed earlier, the mechanistic role of AID/APOBEC family enzymes in demethylation is still unclear. However, growing evidence continues to show how these deaminating enzymes play a direct role in immune development and oncogenesis. As with the DNMT and TET enzymes, this effect appears to be in both myeloid and lymphoid lineage cancers.

In diffuse large B‐cell lymphoma (DLBCL), a recent study has shown that expression of the activation‐induced cytidine deaminase (AICDA) gene that encodes AID is a driver of global methylation heterogeneity and hypomethylation, the features that are correlated with inferior clinical outcome.^179^ Another study investigated the methylation levels of the promoter region of the Fanconi anemia complementation group A (FANCA) gene, which is highly expressed in DLBCL. By correlating AID with recruitment of TET2, subsequent demethylation of the promoter, and increased FANCA gene expression, the complex but significant synergy between AID and TET2 is highlighted, mirroring the similar process discussed earlier that is seen in HSC.^159^, ^180^ Coupled with the findings of Lio et al showing TET enzymes as directly modulating the expression levels of AID through the “AICDA superenhancer,” the framework for an inherent feedback loop appears in place.^158^

As the prognostic and therapeutic value of tumor mutational burden becomes much clearer in both solid and liquid tumor types, the mutagenic role of the deaminase class enzymes beyond AID remains an important question.^181^, ^182^ For example, APOBEC activity defines 3 of 9 mutational signatures identified from over 1000 tumor genomes.^183^

APOBEC3A, significant for its ability to deaminate 5mC, appears to have an important role in oncogenesis. An enrichment of mutational motif specific to APOBEC3A compared to other APOBEC family members has been defined as an “A3A‐like” signature; notably, this signature can be distinguished from other APOBEC enzyme signatures in multiple cancer types with high levels of APOBEC‐driven mutations.^184^ A recent study with potentially significant therapeutic relevance found that the increased APOBEC3A contributes to mutational burden and drives the development of “passenger hotspot mutations” or mutational red herrings that have been mischaracterized as driver mutations.^45^ While many cancer types with APOBEC mutational signatures are solid tumor types, such as breast cancer, the similar role of APOBEC3A has also been shown in a liquid tumor, AML.^185^ Furthermore, an apparently conserved group of AMLs has been identified with high levels of APOBEC3A expression. With subsequent in vitro cell culture studies demonstrating sensitization to DNA replication checkpoint inhibition, this AML outlier group defined by APOBEC3A expression may serve as an important link between clonal hematopoiesis, malignant transformation, and therapeutic approach.^186^

### 7.3 Thymine DNA glycosylase

Conditional *Tdg* inactivation in Apc‐Min mice results in the increased frequency of small intestinal adenomas. Interestingly, this increase occurred in female mice, consistent with TCGA data showing that among colorectal cancer (CRC) cases, the lowest quartile of *TDG* and *APC* expression is associated with an excess of female cases.^187^ As TDG has known interactions with an estrogen receptor, coupled with the fact that women with Lynch syndrome carry a higher lifetime risk of CRC, the connection between the estrogen hormone and colorectal oncogenesis may involve the demethylation machinery.^188^ In the clinical literature, a recent case with a series of colorectal cancer patients identified a nearly fourfold risk increase in the patients carrying a SNP in TDG, rs4135113.^189^ Notably, this G199S variant was shown to induce DNA double‐strand breaks and cellular transformation in cultured cells.

A case of pediatric rectal cancer was reported in the setting of constitutional PMS2 deficiency, one of the Lynch syndrome‐associated MMR genes. A heterozygous missense TDG mutation was noted in the tumor and subsequently confirmed by IHC to result in reduced TDG protein levels. Accordingly, the tumor DNA contained a number of C‐to‐A transition mutations, consistent with the mutagenesis pattern through the 5mC deamination.^190^ Although pediatric colorectal cancer (CRC) is a very rare disease, with a reported incidence of 1 in 1 million, its biology has notable differences from adult CRC; as such, this case may be further evidence of a role for TDG and active demethylation as a cancer phenotype modifier, as opposed to a singular tumor suppressor role seen with TET2. To this point, another case series investigated TDG expression and gene methylation patterns in patients carrying a TP53 SNP that was associated with a form of Li‐Fraumeni syndrome. Referred to as the “Brazilian variant,” the pR337H mutation was discovered as a founder mutation in Brazil and has been associated with the increased incidence of adrenocortical carcinomas (ACC) in children.^191^ Interestingly, increased TDG expression was observed in the ACC patients with the presence of the germline pR337H mutation, further suggesting a possible endocrine/hormonal relationship between TDG and cancer.^192^

We have recently identified TDG as an anticancer target in melanoma. *TDG* knockdown induced cell cycle arrest and killing of melanoma cell lines^54^; cells that escaped these two fates accumulated >4n DNA content and underwent senescence.^54^ Concomitantly, gene set enrichment analysis of *TDG* knockdown melanoma cells revealed the enrichment of E2F target genes, which are known to be involved in cell cycle progression and DNA replication and repair.^54^ Unexpectedly, upregulation of transcripts was also found, encoding immune regulators, secreted cytokines and proteases belonging to the inflammatory and immune response.^54^ This suggests that the normal function of TDG suppresses inflammatory responses, presumably through modulation of DNA methylation levels.

### 7.4 MBD4

Similar to *TET1*, *MBD4* was discovered to have a foundational role in linking cytosine methylation with oncogenesis. Early clinical reports showed a strong correlation between MMR deficiency and secondary *MBD4* mutations in Lynch syndrome spectrum cancers (CRC, endometrial, and pancreatic cancer).^28^, ^193^, ^194^, ^195^, ^196^ Subsequent research highlighted the epigenetic significance of *MBD4* as protecting against 5mC transition mutations to thymine, specifically through the binding of *MBD4* to 5mC deamination products as well as to MLH1 protein directly.^25^, ^197^, ^198^, ^199^, ^200^ These studies are in hindsight the first to link high mutational burden tumor types with epigenetics, with CRC serving as the most popular model. Interestingly, a recent case was reported of early onset rectosigmoid cancer with a germline heterozygous *MBD4* mutation in the absence of Lynch syndrome or other germline colorectal cancer predisposing mutation.^201^ Compared to the aforementioned case of pediatric rectal cancer with heterozygous TDG mutation, these cases appear to highlight the similar but nonredundant features of the 5mC maintenance enzymes, which have been established at both biochemical and model system levels.

More recent clinical studies have presented the significance of *MBD4* as a “cancer phenotype modifier,” including the hematopoietic system. A recent article by Sanders et al described how the accumulation of C‐to‐T transition mutations due to germline loss of MBD4 resulted in the specific progression of clonal hematopoiesis to the early onset of AML.^202^ Perhaps most significantly, the presence of germline *MBD4* mutations in uveal melanoma reintroduced the clinical significance of mutational burden in the dawning era of immunotherapy. In addition to two independent reports demonstrating significant “outlier” responses of uveal melanoma to pembrolizumab in the setting of germline *MBD4* mutations, the direct mechanism of pathogenesis also has begun to be uncovered.^203^, ^204^, ^205^ Indeed, a recent comprehensive analysis of *MBD4* mutations in uveal melanoma confirmed the role of MBD4 as a tumor suppressor in line with Knudson's two‐hit hypothesis, and convincingly joined *BAP1* as the only identified predisposing genes for uveal melanoma.^206^ Future studies may reveal whether the mechanism of tumor suppression by *MBD4* is through addressing spontaneous deamination of 5mC or through a more complex system involving AID/APOBEC enzymes.

## 8 CURRENT AND POTENTIAL ROLE FOR METHYLOME ALTERING THERAPY: DNA HYPOMETHYLATING AGENTS

With such a large physiological investment in maintaining DNA methylation homeostasis in normal cells, and the risk of oncogenesis when disrupted, a natural question would be: can modulating the DNA methylation status be a good therapeutic approach for cancer? The answer appears to be an unequivocal yes, as supported by the efficacy of hypomethylating (demethylating) drugs in specific contexts of cancer.

Decitabine (5‐aza‐deoxycytidine, tradename Dacogen) was first synthesized in 1964. By substituting a nitrogen for the five position carbon in cytosine, the so‐called “azanucleoside” incorporates into DNA and forms a complex with DNMT enzymes; the complex can prevent post‐enzymatic release causing degradation of the enzyme.^207^ Decitabine entered the research consciousness after being demonstrated to have anticancer properties in the 1980s. After a series of trials over the 1990s, which demonstrated its therapeutic potential in AML, pivotal trials using lower doses, as opposed to maximally tolerated doses, showed the differentiation and antiproliferative effects of this drug through hypomethylating effects. By 2006, decitabine was approved for MDS and CMML.^208^, ^209^

The beginning of the past decade was marked by mixed opinions regarding decitabine as a treatment for AML. A phase III trial of decitabine vs supportive care or cytarabine (treatment choice) in patients >65 years of age reported an improved complete remission (including without platelet recovery) of 17.8% with decitabine vs 7.8% in controls, and a nonsignificant increase in the primary analysis of median overall survival to 7.7 months from 5.0 months. As elderly AML patients have poorer outcomes and are often ineligible for standard induction therapy, this study was a notable investigation into alternative approaches. The FDA ultimately did not approve decitabine for AML in elderly patients, whereas its counterpart in Europe approved it for de novo or secondary AML with poor or intermediate risk cytogenetics.^210^

Over the past 5 years, the therapeutic role of hypomethylating agents has been developed in combination therapy. Landmark studies led by DiNardo et al showed that when hypomethylating agents were used in combination with venetoclax, a BCL‐2 inhibitor, a remarkable increase in complete remission (73%) and overall survival (17.5 months) was achieved.^211^, ^212^ Whereas these studies were nonrandomized and open label, a randomized double‐blind phase 3 trial comparing decitabine in combination with venetoclax vs decitabine with placebo is currently ongoing (NCT02993523).^213^

Several reports have alluded to the potential for hypomethylating agents in combination therapy beyond de novo AML. In one report of therapy‐related AML in patients with advanced solid tumors, the complete remission rate of the combination of venetoclax and decitabine was comparable to the rates in de novo AML. While therapy‐related AML is uncommon, it is associated with aggressive cytogenetic features, and is frequently disqualified from clinical trials.^214^

Hypomethylating agents have intriguing and theoretical potential for combination therapy. Hypomethylation has an established effect of endogenous retroviral activation and expression of other anti‐tumor genes, with an overall immune response that has the potential to convert immunotherapy resistant AML to sensitive one.^215^ A nonrandomized open label study investigating azacitadine combined with nivolumab in relapsed/refractory AML reported improved remission rates (22%), compared to the reported rates of 10% to 16% for hypomethylating agents used as single‐agent therapy.^216^ Currently, there are five ongoing clinical trials investigating combination immunotherapy with hypomethylating agents for AML. For more detail regarding immunotherapy for AML, with and without hypomethylating agent combination, readers are referred to recent reviews.^215^, ^217^ Combination hypomethylating agents and immunotherapy outside of AML is also being investigated, at both the in vitro and the clinical level. Patient‐derived xenograft studies on microsatellite stable colorectal cancer, which is less sensitive to immunotherapy than the microsatellite instability counterpart, showed an improved response to the programmed death‐1 (PD‐1) blockade due to the increased expression of antigen presentation‐related genes.^218^ In a very recent clinical report, combining decitabine with camrelizumab, an anti‐PD‐1 antibody, demonstrated significantly higher complete remission rates in relapsed/refractory classical Hodgkin lymphoma compared to camrelizumab alone.^219^

## 9 CONCLUSIONS

As summarized in this review, multiple epigenetic mechanisms modulate cytosine within the genome to produce significant effects on development, immune function, and oncogenesis. As studies continue to demonstrate the effects of cytosine methylation on each of these processes, the opportunities to apply hypomethylating therapies increase. Similarly, as the relationships between immune maturation and cancer are strengthened, the potential for aberrant deamination to play a significant role has begun to emerge. By combining the perspectives of physiological C/5mC modification and the processes of deamination and demethylation, the potential for novel therapies as well as the optimization of therapies that are currently available will likely be more effectively realized.

## CONFLICT OF INTEREST

The authors declare no conflict of interest.

## AUTHOR CONTRIBUTIONS

**Rahul Prasad**, **Timothy J. Yen**, and **Alfonso Bellacosa**: Conceived and wrote the review. **Rahul Prasad** and **Alfonso Bellacosa**: Prepared the figures. **Rahul Prasad**: Prepared the tables.

## Data Availability

Data sharing not applicable to this article as no datasets were generated or analyzed during the current study.
